# Cognitive dysfunction and associated factors in patients with chronic schizophrenia

**DOI:** 10.4103/0019-5545.55936

**Published:** 2005

**Authors:** Latha Srinivasan, R. Thara, S.N. Tirupati

**Affiliations:** * Psychologist, Hunter New England Area Health Service, Australia; ** Director, Schizophrenia Research Foundation (India), Chennai 600101; *** Conjoint Associate Professor, Faculty of Health, The University of Newcastle, Callaghan, NSW 2308, Australia

**Keywords:** Cognitive dysfunction, schizophrenia

## Abstract

**Background:**

Deficits in neurocognitive function are a hallmark of schizophrenia. They are associated with clinical manifestations and the course of the illness. A study of cognitive dysfunction in Indian patients with schizophrenia is of significance in view of a more benign course and outcome of the illness in this region.

**Aim:**

To study cognitive deficits and associated factors in patients with chronic schizophrenia and compare them with those in the normal population.

**Methods:**

We compared 100 patients with chronic schizophrenia with 100 matched normal controls on multiple measures of attention, executive function and memory.

**Results:**

Compared to normal individuals, patients with schizophrenia performed poorly in all cognitive tests. Cognitive deficits in patients were related to gender, education, age, duration of illness, and presence of positive and negative symptoms.

**Conclusion:**

The neurocognitive profile of Indian patients with chronic schizophrenia resembles those of patients in developed countries.

## INTRODUCTION

Schizophrenia is accompanied by impairments in several domains of cognitive function.^1^ Patients with schizophrenia have been found to perform more poorly than normal controls on tasks of attention, memory, executive function, language, learning and motor control.^2^–^4^ In recent times, cognitive impairment has gained importance in terms of emerging theories on the aetiology and treatment of schizophrenia.^5^

Cognitive impairment in schizophrenia has been found to be related to measures of psychopathology^6^,^7^ and outcome.^8^,^9^ Much research on cognition in schizophrenia has been done in developed countries where the outcome was found to be poorer than that in developing countries such as India. It is of interest to know the degree and nature of cognitive dysfunction in Indian patients with schizophrenia. Studies in India have described cognitive deficits in schizophrenia.^10^,^11^ However, a comprehensive evaluation of deficits in all major cognitive domains, and their relation with demographic and clinical variables, has not been done. We compared cognitive deficits and associated factors in patients with chronic schizophrenia with those of a matched normal population.

## METHODS

The case group was a consecutive sample selected from outpatients attending the treatment and rehabilitation centre of the Schizophrenia Research Foundation (India) in Chennai and comprised 100 subjects (men: 60; women: 40) fulfilling the DSM-IV criteria for chronic schizophrenia. A clinical interview and chart review established the diagnosis. All of them were on antipsychotic drug treatment at the time of evaluation. Subjects between the ages of 18 and 45 years, with at least 10 years of school education, were selected. The control group comprised 100 healthy subjects (men: 60; women: 40) with no current, past or family history of any psychiatric disorder. They were selected from among volunteers by the stratified sampling method and matched with subjects from the study group for age, sex and education. All participants gave a written informed consent after being explained the nature of the study. The cases and controls did not differ significantly in their mean age (33.6 years, SD±8.2 vs 33.9 years, SD±8.1; *t*=0.251) and years of formal education (14.3 years, SD±3.1 vs 13.9 years, SD±2.8; *t*=0.893). The patients were ill for a mean duration of 10.4 years (SD±6.8). The neuropsychological tests done are listed in Table 1.^12^–^16^

**Table 1:** Neuropsychological tests done on the study sample

Test done (subtests)	Function measured
Digit Span Test^12^ (forward and backward)	Span of attention (verbal task)
Visual Memory Span^12^ (forward and backward)	Span of attention (non-verbal task)
Digit Symbol Substitution Test^13^	Sustained attention and speed
Visual Number Scanning Ability Test*(time taken, number/minute)	Visual scanning and attention
Ideational Fluency Test*	Executive function—verbal fluency
Ruff Figural Fluency Test^14^ (unique and perseverative responses)	Executive function—non-verbal fluency
Wisconsin Card Sorting Test^15^	Executive functions and cognitive flexibility
Letter–Number Span test^16^ (correct and longest)	Working memory
Delayed Response Learning Test*	Working memory
Verbal Learning and Memory* (immediate, delayed recall)	Logical memory and learning
Verbal Paired Associate Learning Test^12^ (immediate, delayed recall)	Associate learning (verbal)
Visual Paired Associate Learning Test^12^ (immediate, delayed recall)	Associate learning (visual)
Visual Reproduction Test^12^ (immediate, delayed recall)	Immediate and delayed visual memory

* NIMHANS Battery (unpublished)

### Data analysis

The Statistical Package for Social Sciences (SPSS)^17^ was used for data analysis. The chi-square and *t* tests were applied for univariate analysis. Simple correlation and partial correlation analyses were done to measure the relationship between continuous variables. The variables significant at univariate analysis were entered into classification analysis using the Mahalanobi distant statistic method to identify neuro-psychological tests that differentiated normals from patients.

## RESULTS

The mean scores on the Positive and Negative Syndrome Scale (PANSS)^18^ were 10.2 (SD±3.9) for the positive subscale (PS), 9.6 (SD±3.2) for the negative subscale (NS) and 23.6 (SD±5.7) for the general psychopathology subscale (GS).

### Cognitive deficits

The patients performed significantly poorer than normal subjects on all tests of cognitive functions evaluated— attention, executive function, memory—except the number of perseverative responses on the Ruff Figural Fluency test for executive function, and immediate recall on the Visual Reproduction task of memory (Table 2).

**Table 2:** Comparison of the cognitive functions in patients with schizophrenia and normal controls

Neuropsychological test	Normal subjects Mean±SD	Patients Mean±SD	*t* score
**Attention**			
Visual Scanning—time taken	162.9±47.4	241.9±99.8	7.14*
Visual Scanning—number/minute	23.5±3.4	17.0±4.6	11.37*
Digit Span—forward	10.7±1.3	9.0±2.0	6.88*
Digit Span—backward	9.6±1.6	7.3±1.9	9.29*
Visual Memory Span—forward	10.9±1.5	9.0±2.0	7.08*
Visual Memory Span—backward	9.5±1.18	7.5±2.4	7.26*
Digit Symbol Substitution Test	57.0±10.5	41.0±11.6	10.18*
**Executive function**			
*Wisconsin Card Sorting Test*			
Trials administered	98.0±19.2	116.8±17.9	7.14*
Total correct	70.7±6.83	66.1±16.2	2.65*
Total errors	27.3±15.4	50.8±25.6	7.87*
Categories completed	5.8±0.6	3.8±2.2	8.71*
Trials to complete first category	13.2±5.1	35.7±40.7	5.49*
Perseverative response—total	18.1±12.9	41.1±32.8	6.53*
Perseverative errors—total	16.0±10.6	34.3±24.5	6.87*
Non-perseverative errors—total	11.5±6.4	16.6±10.0	4.31*
Conceptual level responses—total	63.9±6.6	51.6±22.0	5.36*
Failure to maintain set	0.21±0.43	0.84±1.2	4.99*
*Other tests*			
Ideational Fluency	17.6±3.4	12.9±3.4	9.72*
Ruff Figural Fluency—perseveration	6.5±6.4	7.4±7.5	0.88 (NS)
Ruff Figural Fluency—unique responses	56.9±16.7	35.2±16.4	9.28*
**Memory**			
Verbal Paired Association—immediate	21.6±1.7	19.0±3.6	6.43*
Verbal Paired Association—delayed	7.9±0.3	7.3±1.4	4.37*
Visual Paired Association—immediate	15.6±1.9	11.5±5.0	7.75*
Visual Paired Association—delayed	5.8±0.4	4.9±1.2	6.60*
Visual Reproduction—immediate	35.4±3.7	33.1±31.7	0.72 (NS)
Visual Reproduction—delayed	31.5±6.1	25.6±9.3	5.25*
Verbal Learning and Memory—delayed	22.3±1.3	20.1±3.3	6.30*
Visual Learning and Memory—delayed	16.6±3.2	13.5±4.9	5.20*
Delayed Response Learning	15.7±1.3	13.1±3.0	7.98*
Letter–Number Span—correct responses	18.0±2.7	14.9±3.5	6.92*
Letter–Number Span—longest item	5.9±0.7	5.3±1.0	5.10*

* p≤0.01, which is significant

NS: not significant

The step-wise, discriminant function analysis identified 10 tests measuring tasks of attention, executive function and memory which differentiated most between patients and normal controls. The minimum D squared statistic and standardized canonical discriminant function coefficients (SCDFC) of the tests are listed in Table 3. A classification analysis based on the SCDFC of these 10 variables classified 92% of the study population appropriately into their original groups as patients and normal subjects.

**Table 3:** Function analysis of patients with schizophrenia and normal controls (standardized canonical discriminant function coefficient)

Neuropsychological test	Patients with schizophrenia	Normal subjects
**Attention**		
Visual Scanning—time taken	6.593	0.319
Visual Scanning—number/minute	2.585	0.509
Digital Span—backward recall	5.214	0.220
Digit Symbol Substitution Test	6.333	0.341
**Executive function**		
*Wisconsin Card Sorting Test*		
Failure to maintain set	4.545	−0.359
*Other tests*		
Ideational Fluency	3.766	0.391
Ruff Figural Fluency—unique respose	5.538	0.278
**Memory**		
Verbal Paired Association—delayed	5.754	0.248
Verbal Learning and Memory—delayed	6.876	0.228
Visual Learning and Memory—delayed	6.007	−0.324

### Social and clinical factors and cognition

Women performed better than men on only one task: the Visual Paired Associate learning test (mean scores: immediate recall=12.7, SD±3.8 vs 10.7, SD±5.5, *t*=2.06, p<0.05; delayed recall=5.4, SD±1.0 vs 4.7, SD±1.3, *t*=3.03, p<0.01). The years of education did not correlate with age or clinical factors. The age and duration of illness correlated with each other (*r*=0.723, p<0.001) but not with PANSS subscale scores. The three PANSS subscale scores correlated positively with each other at a significance level of 0.01 or less (correlation coefficients: PS with NS=0.280; PS with GS=0.499 and NS with GS=0.461).

Table 4 presents the significant correlations (p<0.05) among scores on cognitive tests with education, age (controlling for duration of illness), duration of illness (controlling for age) and scores on each of the subscales of PANSS (controlling for scores on the other two subscales of PANSS). Increasing age correlated with scores on the Digit Span and Digit Symbol Substitution Tests of attention, Ruff Figural Fluency Test of executive function, and verbal working memory tested by the Letter–Number Span test. More years of education correlated with better performance on tasks of attention, executive function, verbal and visual memory. A longer duration of illness correlated with indicators of executive dysfunction on the Wisconsin Card Sorting Test (WCST) and verbal memory. The positive symptom score was related to deficit on a single test of verbal memory, and negative symptoms with performance on measures of attention, executive function and visual memory. The GS score did not correlate with any cognitive deficit.

**Table 4:** Cognitive deficits and social and clinical factors (correlation coefficients)

	Correlation coefficients (p<0.05)					
	
				PANSS		
				
Neuropsychological test	Age	Education	DOI	PS	NS	GS
**Attention**							
Visual Scanning—time taken		−0.257			0.255	
Visual Scanning—number/minute		0.203				
Digit Span—forward	−0.245	0.344				
Digit Span—backward	−0.269				
Visual Memory Span—forward		0.344				
Visual Memory Span—backward		0.311				
Digit Symbol Substitution Test	−0.305	0.253			
**Executive function**						
*Wisconsin Card Sorting Test*						
Trials administered		−0.380	0.223			
Total errors		−0.400			0.280		
Categories completed		0.373			−0.229	
Trials to complete first category		−0.224			0.199	
Perseverative response		−0.352			0.351	
Perseverative errors		−0.356			0.338	
Non-perseverative errors			0.255		
Conceptual level responses					−0.329
*Other tests*						
Ideational Fluency					−0.412	
Ruff test—perseverative	0.212					
Ruff test—unique responses	−0.213					
**Memory**						
Verbal Paired Association—immediate		0.208				
Verbal Paired Association—delayed						
Visual Paired Association—immediate		0.223			−0.245	
Visual Paired Association—delayed		0.392			−0.213	
Visual Reproduction—immediate						
Visual Reproduction—delayed		0.340		−0.222	−0.333	
Verbal Learning and Memory—delayed			−0.197			
Visual Learning and Memory—delayed		0.286				
Delayed Response Learning		0.439				
Letter–Number Span—correct responses	−0.245	0.478				
Letter–Number Span—longest item	−0.252	0.394			

DOI: duration of illness, PANSS: Positive and Negative Syndrome Scale, PS: Positive subscale, NS: Negative subscale, GS: General psychopathology subscale

## DISCUSSION

### Cognitive deficits in chronic schizophrenia

We did not have any difficulty in using the neuropsychological tests developed in other cultures. The significant level of schooling of patients during which English was one of main languages taught seemed to facilitate their ability to understand and perform on tests that had numerate or verbal tasks. We feel cultural factors had little impact on performance in the neuropsychological tests.

Patients with schizophrenia performed poorly on all tests of cognitive function compared with the normal population matched with respect to gender, age and education. The classification analysis showed that patients with schizophrenia can often be clearly differentiated from the normal population based on their performance on some of the tests of attention, executive function and memory.

### Factors associated with cognitive deficits

Gender differences in cognitive dysfunction have been reported. Males have been found to have more cognitive deficits than females, a trend attributed to the interplay of sex hormones, neurodevelopmental and psychosocial sex differences.^19^ We did not find any major gender difference except for a poorer performance of males on a memory task. Age-related decline across most neuropsychological functions has been demonstrated in schizophrenia.^20^ We found that increasing age was related to poorer performance on tasks of attention, executive function and memory, which has been pointed out to be the result of an ageing brain in patients.

More years of education positively influenced performance on tasks that tested attention, executive function, memory and constructional ability. The duration of formal academic training reflected good pre-morbid functioning, intellectual level and a higher level of information-processing skills in the past. Patients with good education thus did well on cognitive tasks because of this inherent capability. A parallel can be drawn with the influence of education on cognitive changes reported in other neurological disorders.^21^–^23^ Cognitive deficits have been found to remain relatively stable throughout the course of schizophrenia.^24^ We also found that all measures, except two measures of executive function on the WCST and one of verbal memory, were stable over a range of illness duration.

We observed that negative symptoms had a strong association with cognitive dysfunction in all the domains. This finding is in agreement with the results of studies which showed that both positive and negative symptoms were associated with distinct neuropsychological deficits.^25^ Heydebrand *et al*.^26^ observed that negative symptoms were related more frequently to cognitive dysfunction than positive symptoms.

## CONCLUSION

In a group of patients with chronic schizophrenia in India, the nature and degree of cognitive deficits and their relationship to gender, age and clinical factors are comparable with observations made in developed countries. It would be of interest to explore the relationship between cognitive deficits in, and outcome of, schizophrenia among Indian patients, as they have a better outcome than patients with schizophrenia in developed countries.
